# A Trouble Shared Is a Trouble Halved: Social Context and Status Affect Pain in Mouse Dyads

**DOI:** 10.1371/journal.pone.0004143

**Published:** 2009-01-08

**Authors:** Laura Gioiosa, Flavia Chiarotti, Enrico Alleva, Giovanni Laviola

**Affiliations:** 1 Department of Cell Biology and Neuroscience, Section of Behavioral Neuroscience, Istituto Superiore di Sanità, Roma, Italy; 2 Institute of Neurobiology and Molecular Medicine, Consiglio Nazionale delle Ricerche, Roma, Italy; Indiana University, United States of America

## Abstract

In mice behavioral response to pain is modulated by social status. Recently, social context also has been shown to affect pain sensitivity. In our study, we aimed to investigate the effects of interaction between status and social context in dyads of outbred CD-1 male mice in which the dominance/submission relationship was stable. Mice were assessed for pain response in a formalin (1% concentration) test either alone (individually tested-IT), or in pairs of dominant and subordinate mice. In the latter condition, they could be either both injected (BI) or only one injected (OI) with formalin. We observed a remarkable influence of social context on behavioral response to painful stimuli regardless of the social status of the mice. In the absence of differences between OI and IT conditions, BI mice exhibited half as much Paw-licking behavior than OI group. As expected, subordinates were hypoalgesic in response to the early phase of the formalin effects compared to dominants. Clear cut-differences in coping strategies of dominants and subordinates appeared. The former were more active, whereas the latter were more passive. Finally, analysis of behavior of the non-injected subjects (the observers) in the OI dyads revealed that dominant observers were more often involved in Self-grooming behavior upon observation of their subordinate partner in pain. This was not the case for subordinate mice observing the pain response of their dominant partner. In contrast, subordinate observers Stared at the dominant significantly more frequently compared to observer dominants in other dyads. The observation of a cagemate in pain significantly affected the observer's behavior. Additionally, the quality of observer's response was also modulated by the dominance/submission relationship.

## Introduction

Social context markedly affects emotional response in many species [1], [2]. Being with familiar conspecifics, compared with being alone, decreases both behavioral and physiological responses to challenging situations [3]. Although the mouse, a gregarious species, is highly motivated to be in a social condition, social context relevance for emotional response has been scarcely investigated in laboratory rodents [4].

For most mammals, behavior of individuals and their responses to external stimuli are controlled by the microsocial environment, which is also associated with dominant-subordinate relationship between pairs of conspecifics. In these pairs, one animal (the dominant) has learnt to dominate the other animal (the subordinate), which in turn tends to avoid confrontation [5]. From this perspective, dominance and subordination are not to be considered individual traits, but dynamic states relative to the particular group of individuals being considered and the context in which the status is achieved [5]–[8]. In a colony housing model, in which adult male and female mice were mixed, dominant males were more behaviorally active and responded to social interactions with a predominantly sympathetic adreno-medullary pattern; subordinate males were less behaviorally active and predominantly responded with a pituitary adreno-cortical pattern [9]–[11]. Consistently, long-attack-latency (LAL) mice are characterized by a “passive” coping style, while short-attack-latency (SAL) mice show an “active” coping style [12]–[14].

In stable dyads of mice, dominants and subordinates differ on the basis of their behavioral response to pain, too. After only a single agonistic episode, resident mice are hyper-algesic on a hotplate, whereas defeated intruders show a hypo-algesic behavioral response [15]. Similarly, extended exposure to attack is essential to the development of an enduring (opioid-typical) analgesia [16].

Recently, Langford and colleagues [17] reported social modulation of pain in mice as evidence of empathy. These researchers observed that the behavioral response to pain in a mouse is modulated by the presence of a conspecific, and additionally noted that the simple observation of a cagemate in pain alters nociception. Specifically they observed an increase of pain response to acetic acid in mouse dyads when both subjects were pain tested and familiar (siblings or cagemates) compared to individually tested animals. Additionally, the researchers reported that mice did not need to be genetically related, but to cohabitate for at least 21 days in order to show this empathy-like phenomenon. It is worth noting that these findings imply communication of pain from one mouse to the other and that such communication is not used by mice that are strangers, but by mice that are familiar with one another. However, a possible role of the dominance/submission relationship occurring between mouse pairs has not been assessed thus far. The effects of interaction between the social status and the social context on pain response in dyads of mice characterized by a stable dominance/submission relationship must also be investigated.

In our study, we investigated whether behavioral response to pain could be modulated in a dyad of mice characterized by such a stable relationship. On the basis of the aforementioned literature, we asked whether the presence of its own (dominant or subordinate) partner could modulate the behavioral response to pain of a mouse during the formalin test. We selected this experimental paradigm because it allows an accurate and long-lasting investigation of behavioral response to both acute and chronic pain. A first peak of behavioral response to pain (typically licking behavior of the formalin-injected paw) during the first 5 minute interval, reflects the behavioral response to acute pain; whereas the second part of the curve represents a persistent pain [18]. In between the two curves, there is an inter-phase (from minute 5 to 10 after formalin injection) in which paw-licking behavior is almost reduced to zero. Additionally, we needed a test whose length allowed the animals to observe a cagemate in pain for a relatively long time. The formalin test lasts 30–50 minutes; therefore, allowing long enough observation of the animals to detect even a slight modulation of behavior in both the subjects. The two members of a pair could either both undergo formalin injection and be pain tested or one is injected and the other is merely observing the behavioral response of its own companion to pain. Moreover, individually pain-tested animals represented an additional experimental group. In our research we focused on a few selected experimental groups (not necessarily completely consistent with previous literature, [17]) so that we could detect even subtle effects of interaction between the social status and the social context during pain testing on nociception in mouse dyads. Indeed, we added a dimension of relative social status since our experimental setting allowed us to study animals with a dominance/submission relationship. Since there is a large amount of documented literature on the difference between dominant and subordinate mice in behavioral coping strategies towards environmental stimuli [13], [14], [19], we monitored behaviors of dyads characterized by a stable dominant-subordinate relationship. Finally, we analyzed the behavior of the animal that was not injected and not pain tested (the observer) to investigate whether the observation of a cagemate in pain could modulate the observer's behavior.

Under the aforementioned conditions, we observed independent effects of social context during pain testing and social status on nociception in mouse dyads. Indeed, it was not the mere presence of a conspecific, but the observation of this conspecific in pain that modulated the behavioral response to pain (nociception reduction). Nonetheless, our results extend to a markedly different pain experimental procedure (the formalin test), which is the social modulation of pain in mice reported by Langford and colleagues [17]. Further, as expected, subordinates were hypoalgesic compared to dominants. Contrary to our expectations, at least, under present conditions, social context during the formalin test (i.e., being individually tested for pain or when in dyads with either both animals or only one in pain) did not affect differentially the nociception in dominant and subordinate mice (no significant interaction between social status and social context).

## Materials and Methods

### Experimental subjects

100 naïve, adult male mice (8–10 weeks) were the experimental subjects. The study employed CD-1 mice (Charles River, Italy), shipped at 8 weeks of age. On arrival, mice were housed together in Plexiglas (40×20×20 cm) cages, up to 2 unfamiliar mice per cage. A wire-mesh partition divided the cage in two portions so that each animal, matched on body weight (±1 g)(mean body weight = 38.00 g), had its own territory in the cage. Both animals had access to water and food *ad libitum* and independently of each other. The vivarium was temperature-controlled and maintained under a 12:12 h light/dark cycle (lights on at 07:00 h). Systematic daily observations and evaluations yielded 35 dyads in which there were clear and stable dominant-subordinate roles for the constituent individuals. Mice underwent a standard test of pain responses to subcutaneous formalin (1% - a relatively mild concentration) injection. Mice were administered the pain test either Individually (IT-individually tested, dominants: n = 9; subordinates: n = 9) (i.e., alone) or as a dyad. Dyad tests were run with both mice injected (BI, dominants: n = 9; subordinates: n = 9) or with only one mouse injected (OI, dominants: n = 9; subordinates: n = 8). Therefore the experiment involved a 2 (dominant vs. submissive) by 3 (IT, OI, BI) factor design, which is described in detail in the following method sections.

All use of animals complied with the Legislative Decree 116/92 guidelines, which have been implemented in Italy by the European Directive 86/609/EEC on laboratory animal protection and experimentation.

### The sensory-contact-modified model

For our experimental purposes, we used and modified an ethologically oriented model of chronic psychological stress based on the natural behavior of male mice, i.e., acquiring and defending their territory [8], [20]. In this paradigm, stable dyads of mice live chronically in sensory contact and physically interact on a daily basis. Briefly, two unfamiliar mice first were housed in a cage separated by a wire-mesh partition for 24 hours. The partition was removed daily (for a total of 6 days) until a clear and stable dominance/submission relationship was achieved. Each day the two animals were allowed to interact freely for 10 minutes. Multiple attacks were allowed. As a rule, if the display of intense aggression provoked wounds, the interaction was interrupted by lowering the partition. These interruptions though, took place very rarely. Physical interactions were interrupted as soon as fights escalated. The dyad was considered stable when one of the two mice achieved the dominant social rank (i.e., for 3 consecutive days the dominant and subordinate roles did not change). The status of “dominant” was considered to have been achieved by a mouse when, during a whole session and in at least 3 subsequent ones, it attacked its partner without ever being attacked; conversely, a mouse was considered to become “subordinate” when, during a whole session and in at least 3 subsequent ones, it was continuously attacked and defeated by the partner, without ever attacking it and showing fully defensive and submissive behavior [see, 19], [21]. 15 dyads were excluded from the study because the dominance/submission relationship was not defined by the end of the week of agonistic encounters.

### Nociceptive assay

The formalin test, which has been previously described in considerable detail [22], [23], took place 24 hours after the last physical interaction. Briefly, in the formalin test, 1% formalin (20 microl) was injected into the plantar surface of the right hind-paw using a 25 microl Hamilton microsyringe with a 30-gauge needle. We opted for a low dose of formalin (1%) as opposed to a higher dose of formalin, such as 5%, since one of the aims of our study was to detect even slight modulations of pain sensitivity induced by the observation of another conspecific in pain. Mice were standing on a glass floor within Plexiglas observation cylinders (25 cm diameter; 22.5 cm high). Mice were habituated to these cylinders with the other mouse to be tested concurrently for 30 minutes before the formalin injection. A wire-mesh partition divided in half the cylinder in order to avoid physical interactions between the two animals, but it allowed both animals to maintain sensory contact with each other. The mice were briefly removed, injected, and replaced in the cylinder; in conditions where both mice received an injection, these occurred within 40 seconds of each other. A video camera positioned below the transparent floor recorded the experimental session (50 minutes post-injection). Experiments took place between 12:00 and 16:00. The observational period was divided into 10 blocks of 5 minutes each, and within each block we recorded the time spent in biting/licking the injected paw. A blinded observer later scored all the videotapes (featuring both one injected-OI and both injected-BI dyads) for quantifiable behaviors other than Licking behavior using an instantaneous sampling procedure. Instantaneous samples were collected every 30 seconds for the duration of the formalin test (100 samples). The sampled behaviors were as follows: Immobility, Grooming behavior (directed to the whole body or to the muzzle), generally Sniffing the environment, Walking, Climbing on the wire-mesh partition, and Rearing behaviors [24]. In order to have a measure of the attention the animals paid to their companions, we also scored the frequency of Staring at the other (the animal is specifically in contact through the wire-mesh partition with its companion, which could be “staring back” or involved in other activities).

### Scoring behavior of the non-injected companion

We scored the companion's behavior using the instantaneous sampling procedure described above. The observational period, in this context, was divided into 4 blocks of 5 minutes, each starting from the last 10 minutes of the habituation period with the cylinder until 10 minutes following the formalin injection of the partner for a total of 20 minutes of observation. Within each 5 minute block an observer, blind to social status of the subjects, sampled the same behaviors listed previously. Since Rearing behavior was proposed originally in rats [25], [26] and more recently was validated in mice as a measure of non-selective attention [27], we combined our data on Rearing and Staring with the other behaviors. We limited such behavioral analysis of the data on “observer mice” to two specific time-points (before and after the formalin injection of the companion), since we were interested in detecting subtle changes in behavior in these subjects resulting from the observation of the cagemate in pain. A difference in the behavior before and after the injection of the companion would have revealed that the observer perceived a change in the companion.

### Statistical analysis

The results of Paw-licking behavior were analyzed with a 3-way ANOVA for repeated measures (10 blocks) to test the effects of social status (dominant vs. subordinate) and social context (individually tested-IT, one injected-OI, both injected-BI). All the tests that we used were two-tailed tests. When significant differences were detected, we conducted separate 2-way ANOVAs (social status and social context) for each time block to clarify the nature of the group differences. Data collected by means of the instantaneous sampling procedure were analyzed with a 3-way ANOVA for repeated measures (10 blocks) to test the effects of social status (dominant *vs.* subordinate) and social context (individually tested-IT, one injected-OI, both injected-BI). We performed a MANOVA on data on observer mice, considering Grooming directed to the whole body and to the muzzle, the sum of Staring at the other and Rearing, generally Sniffing the environment, Walking and Climbing on the wire-mesh partition behaviors. Immobility and Paw-licking behaviors were not included in the MANOVA, since observer mice rarely displayed these behaviors. Afterwards, data on the companions' behaviors were analyzed with a 2-way ANOVA for repeated measures (4 blocks) to test the effects of social status (dominant vs. subordinate). The *post hoc* analysis was run with the use of the Tukey test. In every case a criterion α = 0.05 was adopted.

## Results

### Licking behavior

As expected, a 3-way ANOVA for repeated measures detected a main effect of time on Paw-licking behavior revealing the characteristic biphasic curve of the formalin-induced behavioral response (F_(9, 423)_ = 35.819; p<0.0001). Classically, a first peak of Licking behavior during the first 5 minute block reflects the behavioral response to acute pain, whereas the second part of the curve represents persistent pain. In between these two curves, there is an inter-phase (from minutes 5 to 10) in which Licking behavior is almost reduced to zero.

**Figure 1:**
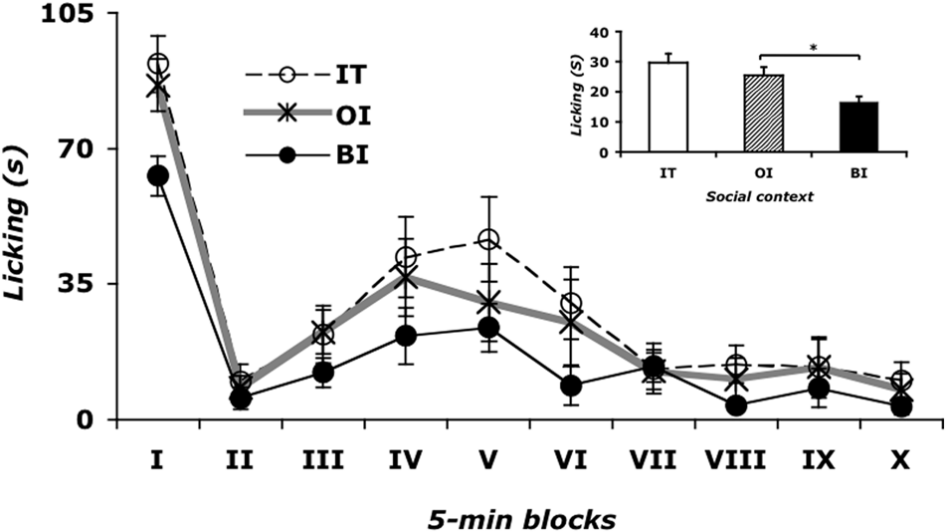
Effect of social context on Paw-licking behavior during the formalin test. The observation period is divided into 10 blocks of 5 minutes each. Data are mean±SEM. n = 16–18. Social context: IT = individually tested; OI = one injected; BI = both injected. Inset of Figure 1. Effect of social context on time spent in Paw-licking behavior induced by a formalin injection. *p<0.05, OI vs BI. Data are mean±SEM. n = 16–18.

There was a significant effect of social context on Paw-licking behavior (F_(2, 47)_ = 6.985; p<0.005) (inset of Fig. 1). The *post hoc* analysis revealed that the OI subjects did not differ from IT mice. Remarkably, BI subjects exhibited half as much Paw-licking behavior as the OI group (BI vs OI, p<0.05).

Since time was significant on nociception (see Fig. 1), we conducted smaller 2-way ANOVAs on Licking behavior. During the first phase of formalin-induced effects, social context was significant (F_(2, 47)_ = 6.985; p<0.005): again OI mice were more sensitive to nociception than BI mice (p<0.05). Similarly, the second phase of the formalin induced behavioral response (from the second to the tenth block) showed a significant effect of social context on persistent pain (F_(2, 47)_ = 5.048; p = 0.01), with a reduction of nociception in the BI condition compared with pain levels in the OI group.

**Figure 2:**
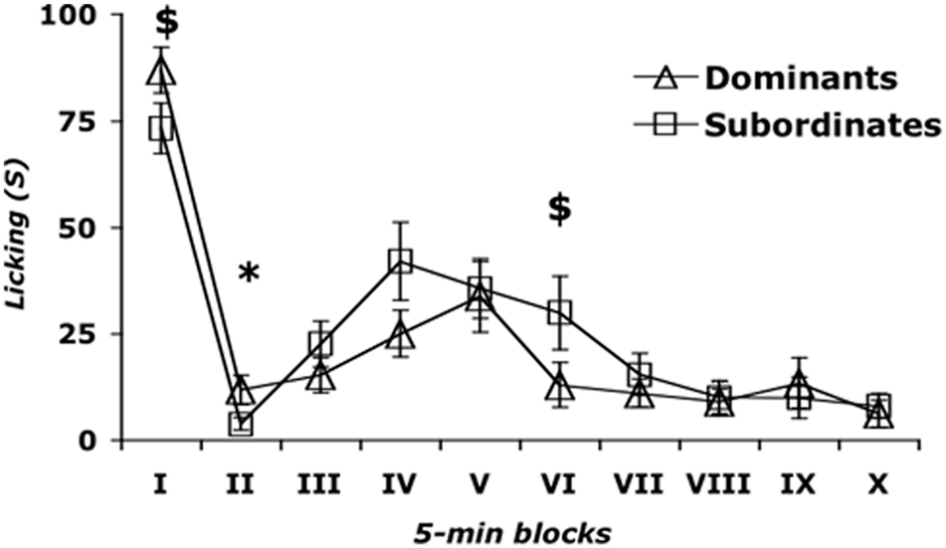
Effect of interaction between social status and time on Paw-licking behavior induced by formalin injection. The picture shows the biphasic curve of formalin-induced effects on licking behavior. Accordingly to literature, a first peak of Paw-licking behavior during the first 5 minute interval reflects the behavioral response to acute pain. The second part of the curve is representative of persistent pain. In between the two peaks, Licking is almost reduced to zero in the interphase (from minute 5 to 10). *p<0.05, dominants vs subordinates during block II; $p<0.10, dominants vs subordinates during block I and VI). Data are mean±SEM. n = 26.

The status by time interaction (Fig. 2) approached statistical significance (F_(9, 423)_ = 1.740; p = 0.08), with dominant mice being more sensitive to formalin than subordinate mice during the first phase (status: F_(1, 47)_ = 3.182; p = 0.08) and the interphase (status: F_(1, 47)_ = 4.056; p = 0.05) respectively; in contrast, the opposite profile was observed during block VI of the late phase of the formalin effects (status: F_(1, 47)_ = 3.218; p = 0.08).

Data analysis did not indicate a significant effect of interaction between social status and context on Paw-licking behavior.

### Instantaneous sampling of mouse dyads

Quantifiable behaviors other than Paw-licking behavior for both OI and BI dyads were scored by means of the instantaneous sampling procedure, while injected mice were being tested for behavioral response to pain induced by formalin. The very first thing that we noticed after the early phase (the first 5 minute block, characterized by an intense display of Paw-licking behavior) was the occurrence of a classical “displacement” activity, i.e. Grooming behavior. Interestingly, mice were progressively more involved in this self-directed activity (time: F_(9, 279)_ = 3.033; p<0.005), in particular in the second 5 minute block, than later on (data not shown). Furthermore, a significant main effect of status appeared (F_(1, 31)_ = 7.118; p<0.05), with subordinate mice grooming themselves more often (by at least a 50%) than dominants.

**Figure 3:**
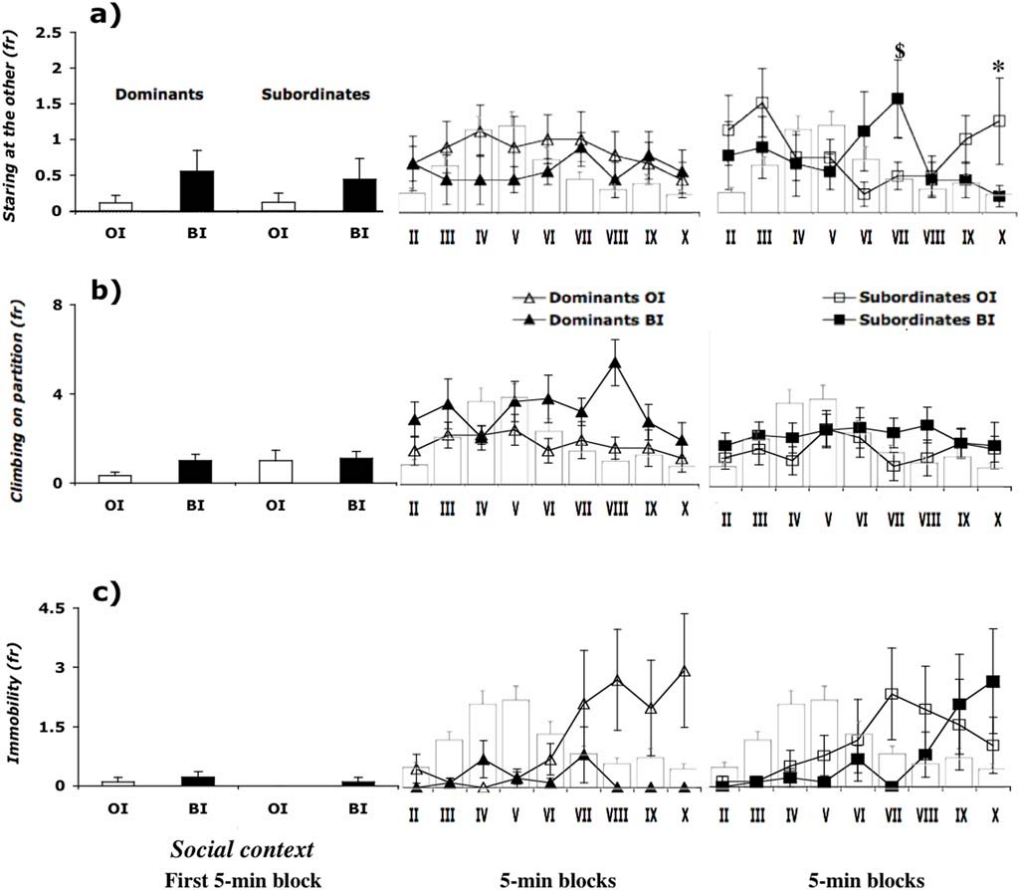
A, Effect of the social status by social context by time interaction on frequency of Staring at the other during the formalin test. $p<0.10 BI subordinate vs OI subordinate in VII block; *p<0.05 BI subordinate vs OI subordinate in block X. B, Effect of the social status by social context by time interaction on frequency of Climbing on the wire-mesh partition during the formalin test. C, Effect of the time by status by social context interaction on frequency of Immobility during the formalin test. On the background it is represented the classical time-course profile of formalin-induced behavioral response (see also legend of Fig. 2). Data are mean±SEM. n = 8–9. Social context: IT = individually tested; OI = one injected; BI = both injected.

The levels of attention, as measured by Staring at the other behavior, increased after the first 5 minute block (F_(9, 279)_ = 1.994; p<0.05) (Fig. 3A). There was a significant effect of the status by social context by time interaction on this behavior (F_(9, 279)_ = 1.972; p<0.05). In the BI condition, subordinate mice were more often found Staring at the other than dominants with a significant peak at block VII, whereas the dominants showed a more constant profile over the session. In contrast, in the OI condition, subordinates and dominants showed a similar profile for this behavior. Moreover, BI subordinate mice were more often interested in their partner's activities in the VII and the X block compared with OI subordinates (p<0.05), whereas they did not differ in other time blocks. Furthermore, dominants from both the OI and BI condition had similar profiles.

Regardless their social status, OI mice were less often observed Climbing on the wire-mesh partition (social context: F_(1, 31)_ = 4.900; p<0.05) compared to BI (Fig. 3B). All mice spent more time on the wire-mesh partition after the first 5 minute block (time: F_(9, 279)_ = 3.805; p<0.005). As the session progressed, their Walking levels decreased (F_(9, 279)_ = 2.372; p<0.05) (data not shown) and they remained in Immobility more and more (F_(9, 279)_ = 4.575; p<0.001) (Fig. 3C).

The effect of the time by status by social context interaction was significant on Immobility (F_(9, 279)_ = 2.326; p<0.05) (Fig. 3C). In the BI condition, subordinate mice showed a temporal profile for Immobility frequency characterized by very low levels, which increased only at the very end of the pain test. In contrast, the dominants' levels were almost reduced to zero through the whole experimental session. Moreover, BI subordinates were more likely to be immobile than dominants. In contrast, in the OI condition, subordinates and dominants' profile for Immobility were similar.

Sniffing behavior significantly increased through time blocks (F_(9, 279)_ = 2.160; p<0.05) (data not shown).

### Companions' behavior

**Figure 4:**
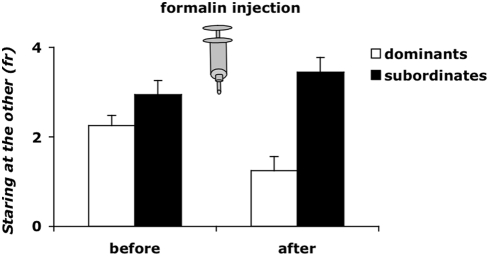
Effect of the social status by time interaction on frequency of Staring at the other by the observer mice (either dominant or subordinate) during the ten minutes before and after their companion was formalin injected, therefore ten minutes before and after the onset of the pain response. Data are mean±SEM. n = 16–18.

We monitored the spontaneous behavior of the non-injected animal (the observer) during the ten minutes before and after their companion was injected with formalin and pain tested (OI condition). Consistent with traditional and classical literature, MANOVA revealed a difference between dominant and subordinate observers, which remained approximately constant before and after formalin injection (main effect of Status: Wilks' lambda = 0.0893, df = 1,15, p = 0.0001; interaction Status×Time: ns). Moreover, MANOVA revealed an almost significant difference in the overall level of activity between pre- and post-injection (main effect of Time: Wilks' lambda = 0.3944, df = 1,15, p = 0.0909). The results from multivariate analysis were consistent with results from univariate analysis. Indeed, dominant observer mice were three times more often involved in Grooming behavior directed to the muzzle compared with subordinate observers of other dyads (status: F_(1, 15)_ = 12.541; p<0.005). As shown in Figure 4, subordinate observers spent significantly more time Staring at the companion's behavior than dominant observers in other dyads (status: F_(1, 15)_ = 18.676; p<0.001). Further, this interest remained constantly elevated irrespective of whether or not their dominant companion exhibited pain response behaviors as a consequence of being injected with formalin. In contrast, when the dominant was the observer, it significantly decreased its interest in its subordinate companion after it received formalin and started to exhibit the typical behavioral pain response (status×time: F_(1, 15)_ = 6.973; p<0.05). No significant main effects or interaction affected the remaining scored behaviors.

## Discussion

In our study, social context consisted of two conditions: subjects that were individually pain tested (IT) and subjects that were tested in its partner's presence. In the latter (i.e., in dyad tests), two conditions were also assessed: either one mouse (OI) or both mice (BI) were injected with formalin and assessed for nociception.

The behavioral response to pain did not differ when animals were tested individually (IT condition) or when only one in a dyad was injected (OI condition) and pain tested. Thus, being alone or in the presence of a partner did not affect the behavioral response to pain during the formalin test. Remarkably, when both mice of a dyad were injected (BI condition), their behavioral response to pain in the formalin test appeared significantly reduced compared to that exhibited when only one animal of a dyad was injected (OI condition). Under present conditions, we observed that it was not the mere presence of a conspecific, but the observation of this conspecific in pain that reduced the behavioral response to pain and presumably pain sensitivity. Previous literature reported that social context modulates behavioral and physiological response to a challenging situation, i.e. being with a familiar conspecific, rather than being with a stranger or alone, modifies behavioral response to pain or to a conflict situation [3], [17], [28]. In particular, in an experimental setting similar to ours, Langford and colleagues [17] observed an increase of nociceptive response when both animals of a dyad were injected acetic acid. Their observation of an increased nociception in the writhing test in mouse dyads in which both animals are injected with acetic acid compared with dyads in which only one mouse is injected appears, however, to be antithetic to our results (i.e., a decreased nociception when both mice were experiencing pain compared with dyads in which only one animal was injected with formalin). Indeed, in our study, animals were in a condition very close to one of the experimental conditions described by Langford and colleagues [17]; specifically, the one in which the animals underwent the acetic acid test after one week of cohabitation. It is worth noticing that under this specific condition Langford and colleagues [17] did not observe any significant difference between the behavioral response shown by animals of a dyad in which both animals were injected, and the behavioral response showed by the individually tested animals (i.e., the controls). In our study, mice were pair-exposed to a challenging situation (i.e., the formalin test) after a week of cohabitation. Under these conditions, we observed a decrease of behavioral response to pain in the BI group compared to OI and IT groups. Social modulation of nociception that we observed can be interpreted in the frame of an empathy-like phenomenon as described by Langford and colleagues [17]. Further, we cannot exclude that a sort of “social buffering” phenomenon [29] occurred in our experiment since we observed an amelioration of the behavioral response to pain. Indeed, the formalin test, triggering a behavioral response to a painful stimulus, can be considered a sort of acute stressor. Signals from the cagemate might have mitigated the behavioral response to formalin in our study. Furthermore, we added a dimension of relative social status since our experimental setting allowed us to study animals with a dominance/submission relationship. Indeed, a basic difference between our study and Langford and colleagues' [17] is that our animals were neither siblings nor total strangers. Further, in our study, mice had been cohabitating for a week at the time of testing, which was characterized by an emotionally intense dominance/submission relationship. Therefore, a difference in housing conditions (clear dominance/submission relationship established during the week preceding the pain test *vs.* group housing) and/or pain testing procedures (formalin test *vs.* acetic acid test) might be at the basis of this apparent discrepancy. However, if we exclude the condition in which animals were complete strangers, it is interesting to note that our results are consistent with the results in Langford and colleagues [17]; when the pain response of animals individually tested did not differ from that exhibited by animals experiencing pain in the presence of their non-injected and thus, non-suffering companions. Taking into accounts all these considerations, our results, therefore, confirm and extend to a quite different pain experimental procedure (the formalin test) the social modulation of pain in mice reported by Langford and colleagues [17].

The two phases of the formalin test are thought to represent respectively behavioral response to acute and to inflammatory pain induced by formalin injection. Quite interestingly, a time-course profile appeared in the social context effect, indicated by the fact that the BI *vs.* OI difference was significant during the first phase of the formalin test; whereas, it was slightly reduced later on during the late phase representing chronic pain. Although the reduced nociception in the BI group, in which both animals were injected with formalin, could be ascribed to the simple fact that the animals were distracted by their companion in pain, it is worth noting that social context significantly affected nociception, mainly in the very first phase of the formalin effects, when the behavioral response to the formalin injection is prominent and quite larger compared with the late phase.

In the present study, we also report an effect of social status on acute pain, so that the repeated defeat experience reduced pain sensitivity (hypoalgesia) in the subordinate subjects. This finding is in line with previous literature reporting hypoalgesia in defeated intruders [15]. There is a great body of literature on the dominant-subordinate differences in behavioral response to pain [15], [16]. Experiments on social conflict in rodents have proved a biologically relevant model of stress-induced analgesia: intruders exposed to resident attacks react with decreased nociception [30], [31]. The long-term analgesic reaction after social conflict may be considered as an adaptive learnt response that brings the subject, more rapidly into a potent protective analgesic state [32]. Analgesia induced by defeat can last a few minutes or more and can be sensitive to opioid antagonists [15], [16] [30], [31]. We did detect a marked social status effect on behavioral response to formalin in the early phase only. Interestingly, the latter reflects a behavioral response to acute pain [23], [33]. Subordinate mice, indeed, appeared to be less sensitive to acute pain compared with dominants. It can be hypothesized that such hypoalgesia is a byproduct of a learning process deriving from the acquirement of social status. From the perspective of the two different phases of the formalin test, our results are in line with previous literature on acute pain, in which subordinates are hypoalgesic compared to dominants [15]. To our knowledge, no previous study reported a status effect on the early acute phase of formalin test in mice, as we report here. On the other hand, we did not observe status differences in the late phase of the formalin test, which should reflect a behavioral response to persistent pain. Neurobiological mechanisms that are thought to be enrolled in chronic nociceptive experience have been shown to differ markedly from those activated by acute pain [16], [18].

Although in our study both social status and social context modulated the behavioral response to formalin, under present conditions we did not observe any significant interactions between social status and social context on nociception induced by formalin. Contrary to our expectations, social context during the formalin test (i.e., being individually tested for pain or when in dyads with either both animals or only one in pain) did not differentially affect the profile for formalin behaviors of dominant and subordinate mice. Future studies using different pain testing procedures will be needed to further evaluatate of the effects of interaction between social context and status on social modulation of nociception.

Social status also affected other behaviors besides the specific Paw-licking response to formalin. Interestingly, subordinate mice were considerably more often involved in self-directed Grooming activity than dominants. However, it should also be noted that, irrespective of social status, all mice started an intense Grooming activity during the interphase (i.e., as soon as “compulsive” Paw-licking behavior dramatically decreases). It is noteworthy that Grooming is one of those behaviors that may be performed in stressful situations as displacement activities [34]. On the other hand, dominants were generally more active, spending less time in Immobility, Climbing more often on the wire-mesh partition of the cylinder and Stared at the companion in a constant way, compared with their subordinate partners. The entire set of behaviors indicates the emergence of a quite different coping strategy between dominant and subordinate mice in a dyad. These observations are consistent with the well-known behavioral coping strategies of dominant and subordinate subjects: the former being more active, with the latter being more passive when compared with each other. Differences in coping strategies associated with the dominance/submission relationship have been reported in previous studies. Maestripieri and colleagues [35] reported that repeated daily interactions of the same pairs of individually housed male mice produced a clear distinction between attacking (dominant) and defeated (subordinate) animals in levels of locomotor activity that also correlated with differences in neurotrophin blood levels (see also [36]). Among the neurotrophins, nerve growth factor is directly involved in pain modulation [37], notably nociception associated with inflammation [38]. As outlined above the different coping strategies also include the ability to modulate pain sensitivity and possibly their neurobiological correlates.

In our study, we were also interested in investigating the modulation of behavior of the cagemate that was not injected (OI condition). Quite interestingly, dominant and subordinate behavioral profiles of the observers (non-injected mice) differed during the pain test of their companions. In dyads in which only the subordinate was injected with formalin, their dominant observer partner displayed consistently higher levels of Self-grooming behavior compared with those exhibited by subordinate observers that in other dyads were exposed to their dominant companion in pain. It is worth noting that Grooming behavior is one of those activities that could be displayed just for mere self-cleaning, or as a substitution activity that reflects a conflict due to a stressful condition [34]. The level of stress perceived when exposed to their partner in pain, seems to vary considerably as a function of social status. On other hand, when the dominant subject was injected with formalin, the subordinate observer devoted constant and elevated time in Staring at its dominant partner. This elevated level of social attention was apparently not modulated by the onset of the pain experience of their dominant partner, which suddenly started after the formalin injection. This picture was especially true during the phase of acute pain, i.e. while its injected companion was exhibiting the highest levels of nociceptive response to the formalin injection. Notably, the whole picture was completely different in the opposite condition (namely, when it was the subordinate to be injected with formalin).

In the wild, the dominant behavioral strategy is regulated by various costs and benefits, depending on species-specific social organization along with specific environmental and social conditions [39]–[41]. In contrast, in laboratory models, at least under present conditions in which the two partner mice were separated by a wire-mesh partition, shared half of the cage space, and had independent and *ad libitum* food and water access (see Methods section), no actual benefits appear evident or are attributable specifically to dominants (no prior or exclusive access to resources). In our laboratory model, we took advantage of forcing selected behavioral responses to an unfamiliar conspecific into unnatural/unavoidable confrontation contexts. Nonetheless, present findings are in line with previous literature on the effects of social status on pain sensitivity and on the social modulation of pain in mice, although an effect of interaction between social context and status on pain modulation was not observed.

Furthermore, this study adds to the literature the first evidence that the observation of a cagemate in pain affected the non-injected observer's behavior. In dyads in which the dominant is the observer of the subordinate in pain, the subordinates were consistently more frequently involved in the Staring at the other behavior, both before and after the formalin injection of their dominant companions, so that a difference in status emerged.

Finally, advances in our evolutionary understanding of factors impacting on different levels of perception of other conspecifics will ultimately provide tools in understanding a variety of empathy disorders.
